# Adrenal insufficiency is a contraindication for omalizumab therapy in mast cell activation disease: risk for serum sickness

**DOI:** 10.1007/s00210-020-01886-2

**Published:** 2020-05-06

**Authors:** G. J. Molderings, F. L. Dumoulin, J. Homann, B. Sido, J. Textor, M. Mücke, G. J. Qagish, R. Barion, M. Raithel, D. Klingmüller, V. S. Schäfer, H. J. Hertfelder, D. Berdel, G. Tridente, L. B. Weinstock, L. B. Afrin

**Affiliations:** 1 Institute of Human Genetics, University Hospital Bonn, Venusberg-Campus 1, D-53127 Bonn, Germany; 2 Department of Internal Medicine, Gemeinschaftskrankenhaus Bonn, Bonn, Germany; 3 Department of General and Visceral Surgery, Gemeinschaftskrankenhaus Bonn, Bonn, Germany; 4 Department of Radiology, Gemeinschaftskrankenhaus Bonn, Bonn, Germany; 5 Center for Rare Diseases, University Hospital Bonn, Bonn, Germany; 6 Medical Office for Internal Medicine, Meckenheim, Germany; 7 Medical Office for Diabetology, Niederkassel, Rheidt, Germany; 8 Malteser Waldkrankenhaus St. Marien, Medical Clinic II, Erlangen, Germany; 9 Department of Endocrinology, University Hospital Bonn, Bonn, Germany; 10 Department of Rheumatology and Clinical Immunology, Clinic for Internal Medicine III, University Hospital Bonn, Bonn, Germany; 11 Institute of Experimental Hematology and Transfusion Medicine, University Hospital Bonn, Bonn, Germany; 12 Marien Hospital Wesel, Wesel, Germany; 13 University of Verona, Verona, Italy; 14 Department of Internal Medicine, Washington University School of Medicine, St. Louis, MO 63141 USA; 15 Armonk Integrative Medicine, Hematology/Oncology, Purchase, New York, NY 10577 USA

**Keywords:** Omalizumab, Mast cell activation disease, Serum sickness, Complement activation, Serum sickness therapy

## Abstract

Omalizumab is an effective therapeutic humanized murine IgE antibody in many cases of primary systemic mast cell activation disease (MCAD). The present study should enable the clinician to recognize when treatment of MCAD with omalizumab is contraindicated because of the potential risk of severe serum sickness and to report our successful therapeutic strategy for such adverse event (AE). Our clinical observations, a review of the literature including the event reports in the FDA AE Reporting System, the European Medicines Agency Eudra-Vigilance databases (preferred search terms: omalizumab, Xolair®, and serum sickness) and information from the manufacturer’s Novartis database were used. Omalizumab therapy may be more likely to cause serum sickness than previously thought. In patients with regular adrenal function, serum sickness can occur after 3 to 10 days which resolves after the antigen and circulating immune complexes are cleared. If the symptoms do not resolve within a week, injection of 20 to 40 mg of prednisolone on two consecutive days could be given. However, in MCAD patients whose adrenal cortical function is completely suppressed by exogenous glucocorticoid therapy, there is a high risk that serum sickness will be masked by the MCAD and evolve in a severe form with pronounced damage of organs and tissues, potentially leading to death. Therefore, before the application of the first omalizumab dose, it is important to ensure that the function of the adrenal cortex is not significantly limited so that any occurring type III allergy can be self-limiting.

**Electronic supplementary material:**

The online version of this article (10.1007/s00210-020-01886-2) contains supplementary material, which is available to authorized users.

## Introduction

Omalizumab has become increasingly important in the treatment of diseases (e.g., allergic asthma, chronic urticaria, mast cell activation disease) where there is increased activation of mast cells (Foroughi et al. 2007; Thomson and Chaudhuri 2012; Incorvaia et al. 2014; Stokes 2017; Peterson and Coop 2017; Kavati et al. 2019). This medication has US Food and Drug Administration (FDA) and European Union (EU) approval for treatment of IgE-induced asthma and in chronic idiopathic urticaria. Particularly, in the case of primary systemic mast cell activation disease (MCAD), which in many cases is challenging to treat, omalizumab has proven useful in decreasing symptom intensity (Molderings et al. 2011, further references therein; Zampetti 2018; Broesby-Olsen et al. 2018; Slapnicar et al. 2019; Lemal et al. 2019). Nonetheless, we show here that in certain circumstances, omalizumab may pose a risk for serum sickness. Our clinical observations, a review of the literature including the event reports in the FDA Adverse Event Reporting System, the European Medicines Agency Eudra-Vigilance databases (preferred search terms: omalizumab, Xolair®, and serum sickness) and information from the manufacturer’s Novartis database were used in the present analysis.

The aims of this study are to enable the clinician to recognize when a treatment of mast cell–related disease with omalizumab is contraindicated because of the potential risk of severe serum sickness (i.e., steroid use with resulting adrenal insufficiency) and to report our successful therapy strategy for such adverse event, since no evidence-based guidelines exist for the treatment of severe serum sickness.

### Mast cells, systemic mast cell activation disease (MCAD), and its therapy

Mast cells are hematopoietic tissue immune cells that act both as effector and regulatory cells (Afrin et al. 2016). They play central roles in adaptive and innate immunity (e.g., Cardamone et al. 2016). This versatility is reflected in the myriad of immunologic and non-immune activation stimuli (e.g., by G protein-coupled receptors) resulting in the secretion of a large number (> 1000) of pre-stored mediators (e.g., histamine, tryptase, and numerous de novo–synthesized lipid mediators), chemokines, and cytokines (Ibelgaufts 2019). The profile of such mediators can markedly differ between and within organs/tissues, depending upon the micro-environmental factors and/or the nature of the stimulus (e.g., Marshall et al. 2003).

MCAD (prevalence up to 17% [Molderings et al. 2013; Lyons et al. 2016; Lazaridis and Germanidis 2018]) comprises a heterogeneous group of multifactorial disorders characterized by epigenetic and genetic alterations (somatic and germline mutations) in a variety of genes inducing an inappropriate release of variable subsets of mast cell mediators together with accumulation of either morphologically altered and immunohistochemically identifiable mutated mast cells (systemic mastocytosis and mast cell leukemia) or alternatively, morphologically ordinary mast cells due to impaired apoptosis (mast cell activation syndrome and well-differentiated systemic mastocytosis) (Afrin et al. 2016; Online Resource 1).

MCAD can affect single and multiple systems (i.e., organs and tissues; Theoharides et al. 2015), usually manifesting with symptoms in a subacute or chronic waxing/waning or recurrent manner (Afrin et al. 2017, further references therein). Due to both the widespread distribution of mast cells and the great heterogeneity of aberrant mediator expression patterns, symptoms can involve virtually all organs and tissues; hence, the clinical presentation of MCAD is very diverse (Online Resource 2).

**Fig. 1:**
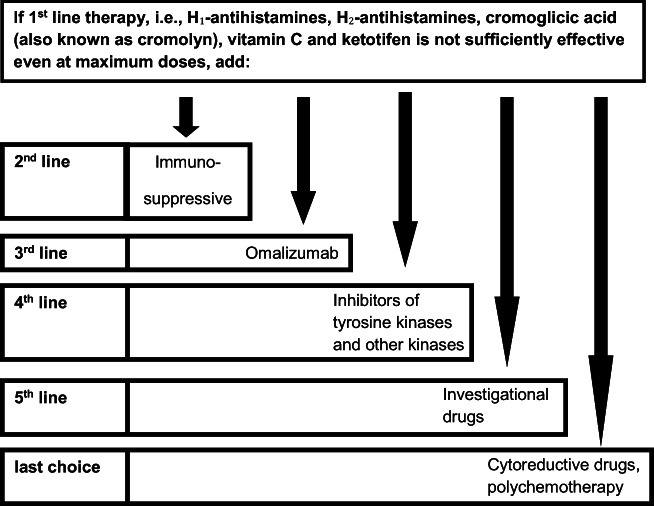
Therapeutic procedure in mast cell activation disease (modified from Molderings et al. 2016)

Currently, there is no cure for primary mast cell disorders. As the dominant feature of MCAD is inappropriate increased and unregulated mast cell activation, treatment invariably involves trigger identification and avoidance, respectively, plus control of mast cell mediator production and action. Generally, less expensive interventions (e.g., histamine H_1_ and H_2_ receptor antagonists, leukotriene receptor antagonists) are tried first, but often symptoms persist. Overall, the medical treatment strategy is a stepwise approach to manage the increased mast cell activity (Fig. 1; for a comprehensive review of the current and potential future treatment options of MCAD, see Molderings et al. 2016).

### Omalizumab

Omalizumab is a recombinant 95% humanized IgG1κ monoclonal antibody (mAb; 5% of murine origin mainly in the Fab fragment; Presta et al. 1993, 1994) which recognizes and binds with picomolar affinity to the third constant domain of the IgE heavy chain (Cε3) of circulating IgE (Vichyanond 2011; Pennington et al. 2016; Davies et al. 2017; further references therein). Cε3 (even more so the Cε3–4 portion) is the docking site that normally binds electrostatically to the α2 subunit of the high-affinity (FcεRI) and low-affinity (FcεRII) IgE receptor on mast cells, basophils, and other cell types. Omalizumab forms immune complexes (ICs) with free IgE and, thus, prevents its interaction with both receptors. An important property of omalizumab is that it neither interacts with cell-bound IgE, nor activates mast cells or basophils (Belliveau 2005). Following subcutaneous administration, omalizumab is absorbed slowly (linearly above doses > 0.5 mg/kg), reaching peak serum concentrations after an average of 7–8 days [Omalizumab, Xolair, label information at www.fda.gov/cder/foi/label/2003/omalgen062003LB.pdf.]. The omalizumab-IgE complexes are cleared via interactions with Fcγ receptors of the hepatic sinusoidal and other endothelial cells of the reticuloendothelial system (Ghetie et al. 1996; Mariani and Strober 1990). Noteworthy, the clearance of the free mAb itself is slow (mean 2.4 ± 1.1 ml kg^−1^ day^−1^), with a terminal half-life (*t*_1/2_) of 26 days (Omalizumab, Xolair, label information at www.fda.gov/cder/foi/label/2003/omalgen062003LB.pdf; Lowe et al. 2009). According to general pharmacokinetic principles, elimination is near-complete after five half-lives of a drug. However, it has been observed that omalizumab may put a disease into clinical remission for up to 6 years after drug discontinuation (Nopp et al. 2007, 2010; Molimard et al. 2014; Bölke et al. 2019), which may imply that the specific ICs remain in circulation long after discontinuation of treatment (Starke P 2016 Clinical Review. BLA 103576 S-5225 - Xolair [Omalizumab]; further references therein). As omalizumab targets a specific component of the immune system, therapy may have the potential to increase the risk of immune disorders. The mAb component of murine origin may induce allergic reactions (for further serious adverse effects, see Online Resource 3).

### Type III hypersensitivity reactions

Type III hypersensitivity reactions (Online Resource 4), also termed Immune Complex reactions, are mediated by IgM and IgG antibodies which react with soluble antigens (including allogenic/chimeric antibodies, such as omalizumab) forming ICs of different sizes (Shmagel and Chereshnev 2009). Serum sickness and serum sickness–like reactions are delayed type III hypersensitivity clinical expressions (Online Resource 4) becoming evident usually 3 to 10 days after exposure to the antigen, when antibodies have been sufficiently generated against the foreign protein and have formed ICs with these circulating antigens. Multimeric antigen-antibody complexes are efficient activators of the complement cascade through its classical pathway. Damage results from the action of cleaved complement anaphylatoxins C3a and C5a (that can be detected by decreased levels of circulating C3 and C4), which, respectively, mediate the induction of granule release from mast cells and recruitment of inflammatory cells leading to tissue damage through phagocytosis by neutrophils and macrophages. Tissue injury typically involve kidney, skin, and mucous membranes (Online Resource 4). The frequency (Online Resource 5) and severity of serum sickness depends on the composition of the antibody, the size of the ICs, and the functionality of the adrenal gland. As long as the adrenal gland is able to produce the amount of cortisol necessary to suppress the inflammatory immune response, serum sickness remains a self-limited disease which will usually resolve upon discontinuation of the offending agent.

The diagnosis of serum sickness is primarily based on patient history, including recent medications but also can be suspected by specific symptoms (Online Resource 4) and laboratory data, including leukocytosis, mild thrombocytopenia, elevated acute-phase proteins such as C-reactive protein (CRP) and Factor VIII, low C3 and C4 complement factors, decreased total hemolytic complement (CH50), and elevated circulating IC, as detected by C1q consumption. Laboratory results may be widely variable and contribute to the diagnosis only if they are positive.

### Use of omalizumab in the treatment of systemic mast cell disease

Omalizumab is an effective therapeutic drug in many cases of MCAD (for references, see Introduction). The initial hope that the risk of AEs would be lower for omalizumab than for other chimeric antibodies, because of humanization procedures and the selective binding with IgE, has not been met (Online Resource 5). In particular, the type III allergy serum sickness has been assumed to be a rare AE of omalizumab therapy. Although its real prevalence can only be roughly estimated, the fact that this AE has been repeatedly reported in various studies with small to medium numbers of patients argues against it being a rare event (Table 1). This assumption is supported by the number of reports in the spontaneous reporting system of the FDA and EMA (Table 1). For other drugs entered in the same databases, it is estimated that only 1% to 10% of all AEs have ever been reported (Rogers et al. 1988; Scott et al. 1987). Whether the same is true of omalizumab-induced serum sickness remains unclear.

**Table 1:** Frequency of occurrence of serum sickness reported in the literature and diagnosed in own patients

Disease treated with omalizumab; reference	Number of pts. treated with omalizumab	Number (%) of pts. in whom serum sickness occurred	Outcome of the serum sickness	Simultaneously applied drugs affecting adrenal gland function
MCAD; own data (Molderings and coworkers)	32	3^a^ (10%)	One pt. died from multiple organ failure	Prednisone > 20 mg/day
			One pt. has irreversible impairment of organs and tissues^b^ after multiple organ failure	Prednisone > 20 mg/day
			One pt. without permanent damages, so far	Prednisone < 10 mg/day
Asthma bronchiale, GI food allergy, MCAD; own data (Raithel and coworkers)	22	1^a^ (4.5%)	One pt. developed myalgia, arthralgia, fever 8 to 15 days after anti-IgE injection; this condition got more and more worse with 3–7 days immobility	
MCAD; Molderings et al. (2011)	4	1^a^ (25%)	No permanent damages	Prednisone > 20 mg/day
Systemic mastocytosis; Jandus et al. (2011)	Case report	n/a	Persistence of symptoms	None
Anaphylactoid reactions; Dreyfus and Randolph (2006)	Case report	n/a	n/a	n/a
Allergic asthma; Berger et al. (2003)	225	0 (0%)		Low-dose inhaled glucocorticoids; doses n/a
Allergic asthma; Pilette et al. (2007)	Case report	n/a	Died from multiple organ failure	Chronic oral glucocorticoid treatment
Allergic asthma; Corren et al. (2009)	3678	0 (0%)		Inhaled glucocorticoids; doses n/a
Allergic asthma; Klyucheva et al. (2013)	15	1 (7%)	No permanent damages	Chronic inhaled glucocorticoids at high doses; 27.7 ± 19.6 mg prednisone/day
Allergic asthma; Harrison et al. (2015)	250	0 (0%)		n/a
Allergic asthma; Chipps et al. (2017)	806	0.1%	n/a	n/a
Allergic asthma; Althin (2018)	38	1 (diagnosis in a second pt. uncertain); /5%)	n/a	n/a
Allergic asthma; Novartis^c^	n/a	> 0	n/a	n/a
Allergic asthma; reported by health care professionals^d^	3478	0.21%	n/a	Inhaled glucocorticoids; doses n/a
Allergic asthma; chronic urticaria, angioedema; Dreyfus and Randolph (2006)	Case report	n/a	n/a	n/a
Chronic urticaria, angioedema; Weiss and Smith (2019)	Case report	n/a	At the time of publication persistence of symptoms	No application of corticosteroids
Chronic idiopathic urticaria; Genentec Inc.^e^	319	1 (0.3%)	n/a	n/a
Chronic spontaneous urticaria; Novartis^c^	412	12 (3%)	n/a	n/a
Chronic spontaneous urticaria; Eapen and Kloepfer (2018)	Case report	1	n/a	n/a
Idiopathic urticaria, angioedema; Kumar (2019)	n/a	1		“Short course of prednisone”; doses n/a
Drug Commission of the German Medical Association 04/04/2018	12,365	67 (0.5%)	n/a	n/a
FDA Adverse Event Reporting System^f^, accessed 09/30/2019	41,617 pts. since 2008	147 (0.4%)	n/a	n/a
European Medicines Agency, Eudra-Vigilance database^g^, accessed 10/05/2019	14,589	87 (0.6%)	n/a	n/a
https://patientsville.com/xolair	5663	279 (4.9%)	n/a	n/a
Novartis, unpublished data	Cases between 01/01/2016–12/31/2016	21 cases; 0.15 pts. per 1.000 pt. treatment years	n/a	n/a

^a^Proven by accidental re-exposure

^b^Kidney, thyroid gland, bursae of both elbow joints and bursae praepatellaris, arthralgia, edema in the lower legs, pulmonary function due to pulmonary embolism, brain dysfunction due to cerebral embolism, damaged muscle fasciae and serous membranes, unstable diabetes, reduced general condition, disabled

^c^https://www.medicines.org.uk/emc/product/4725/smpc

^d^https://patientsville.com/omalizumab/list.htm?ezpage=12

^e^Clinical Trial NCT01287117, study Q4881g, Genetech. Inc.

^f^https://open.fda.gov/data/faers/

^g^https://bi.ema.europa.eu/analyticsSOAP/saw.dll?PortalPages

Thus, it is important to consider several factors before deciding to use omalizumab in MCAD patients. In particular:

#### Patients with normal adrenal function

At present, omalizumab is a third-line treatment option for MCAD (Fig. 1), which in some patients improves symptoms, reduces the number of flares, and strikingly alleviates pain intensity, in particular, in those MCAD patients with pain as a dominant symptom (Molderings et al. 2011; further references therein). Although omalizumab has been reported as a well-tolerated agent (Stokes 2017; Broesby-Olsen et al. 2018), transient mild to moderate mast cell mediator–induced symptoms occurring within several hours after injection have been observed, suggesting possible activation of mast cells, possibly outweighing the desired pharmacological effect of omalizumab (Molderings et al. 2011). This implies that when the triggering of mast cell mediator release (the mechanism of triggering has still to be identified, since the drug is thought to bind only to free IgE) occurs after any of the first three injections, or even becomes more intense from injection to injection, the treatment with omalizumab should be stopped immediately. In the worst case, serum sickness with IC formation, cytokine release, and intense mast cell mediator–induced symptoms may occur after 3 to 10 days; this reaction resolves after the antigen and ICs are cleared. If the symptoms do not resolve within a week, injection of 20 to 40 mg of prednisolone on two consecutive days could be given to stop serum sickness.

#### Patients with complete adrenal insufficiency

Usually, serum sickness would not have such a threatening nature unless three conditions typically found in MCAD can mask and/or turn it into a serious AE:

1. The symptoms of MCAD (Online Resource 2) can be indistinguishable from those of serum sickness. Therefore, an omalizumab-induced serum disease may not be recognized even for a long time in these patients, i.e., until the occurrence of complement-related organ and tissue failures which are less common in MCAD suggests the concomitant presence of serum sickness.

(2) The regular administration of 150 mg or 300 mg omalizumab in 2- to 4-week intervals is more prone to change a usually self-limiting type III allergy into a more serious AE, due to a progressive and significant activation of the complement system showing more persistent and destructive properties.

(3) MCAD patients, especially those with first- and second-line-resistant therapy, aggressive or advanced MCAD, are also treated with glucocorticosteroids at doses (as a rule with prednisone > 20 mg/day) above the Cushing dose (commonly defined as prednisone equivalent > 7.5 mg/day) (Akin 2014; Quintas-Cardama et al. 2006; Valent et al. 2005; Zen et al. 2011; Valent et al. 2012; Afrin et al. 2016). The chronic administration of glucocorticoids in such doses results in a complete loss of endogenous cortisol production by the cells in the zona fasciculata of the adrenal cortex. If serum sickness develops in a MCAD patient treated with such glucocorticoid doses and omalizumab, the serum sickness symptoms would be additionally masked/delayed by the immunosuppressive effect of the exogenous glucocorticoid, without decisively preventing at this dosage the activation of the complement system. When in such a patient the exogenous glucocorticoid dose is tapered off according to the applicable time frame (e.g., Pavlicek 2014), e.g., because the therapy of MCAD is switched to another immunosuppressive agent (e.g., kinase inhibitor), pharmacokinetics indicates a high probability that a flare of serum sickness will occur due to massive complement activation with possible consequent organ failures [as seen with occurrence of Churg-Strauss syndrome (Giavina-Bianchi et al. 2007; Pabst et al. 2008; Ruppert et al. 2008; Jachiet et al. 2016)]. In the absence of a prompt intensive medical treatment and/or misunderstanding of the cause, this flare can be fatal (Table 1). In this situation, besides ensuring support of vital signs, it is crucial to suppress the activation of the complement system with high doses of glucocorticosteroids. There are no evidence-based guidelines for the daily glucocorticoid dose to be used; hence, different daily doses of up to 1 g prednisone are reported in the literature (Pilette et al. 2007; Kumar 2019). We have decided to start probatorily in our patients with prednisone 80 mg/day which turned out as enough for successful treatment, followed by symptom-adapted reductions in prednisone dose, which should take into consideration the peculiarity of omalizumab pharmacokinetics. As said, the average biological half-life of this mAb is about 26 days, which means that only after 5 months (i.e., five half-life periods) would the original compound be eliminated from the body. More importantly, omalizumab ICs may circulate in the body for more than 1 year after the substance has been discontinued (Chang 2000; Tridente 2014; Starke 2015). It is not known whether these ICs are able to sustain serum sickness or other AEs. Since a detection assay of omalizumab-specific circulating ICs is not available commercially, the duration of therapy can only be based on the degree of the symptoms of complement activation when tapering the initial dose of prednisone. However, the differential identification of symptoms is difficult because of their extensive overlap with MCAD symptoms. Of note, the parameters for detecting complement activation (see above) may be initially pathologically altered, but the changes are short-lasting. As for the patients we have treated (Table 1, first row), the time to resolution of severe serum sickness was up to 15 months. At least one patient had significant irreversible disorders. Whether the less severe residual disorders will be irreversible in the second surviving patient remains to be seen.

## Conclusions

Omalizumab therapy may be more prone to cause serum sickness than previously thought. Indeed, in a patient whose adrenal cortical function is completely suppressed by exogenous glucocorticoid therapy for the treatment of the underlying disease (such as MCAD, long-term inhaled corticosteroid therapy of asthma, chronic urticaria, and others), there is a higher risk that serum sickness will be masked and evolve into a severe, potentially fatal form with pronounced damage of organs and tissues. Since the diagnosis of serum sickness is essentially clinical (because the sensitivity of the laboratory parameters is unreliable, and the overlap of serum sickness symptoms with those of the omalizumab-treated underlying disease), recognition of serum sickness disease can be very difficult and often only occurs by chance. If there is a clinical suspicion of correlation of the occurrence of symptoms with omalizumab administration, it may be diagnostically useful to compare the values for the acute phase proteins CRP and Factor VIII determined shortly before the re-exposure with omalizumab and during the symptomatic period after re-exposure. Most importantly, before the application of the first omalizumab dose (and probably other antibodies too), it is necessary to ensure that the function of the adrenal cortex is not significantly limited (which can be excluded by determination of basal and adrenocorticotropic hormone-stimulated cortisol), so that any occurring type III allergy/serum sickness can be self-limiting.

## Electronic supplementary material

ESM 1 (DOCX 24 kb)

ESM 2 (DOCX 26 kb)

ESM 3 (DOCX 25 kb)

ESM 4 (DOCX 29 kb)

ESM 5 (DOCX 28 kb)
