# Supplementary material for: Adrenal insufficiency is a contraindication for omalizumab therapy in mast cell activation disease: risk for serum sickness
Source: Naunyn Schmiedebergs Arch Pharmacol. 2020 May 6;393(9):1573–80. doi: 10.1007/s00210-020-01886-2 (PMC7419348; doi:10.1007/s00210-020-01886-2)
Supplement: Supplementary file 1 — (DOCX 24 kb) [file 210_2020_1886_MOESM1_ESM.docx]

Table 1 Current classification of systemic mast cell activation disease

| **Systemic mast cell activation disease (MCAD)** | | |
| --- | --- | --- |
| Variant | Systemic mastocytosis (SM)  (special case by definition:   - KIT^D816X^ positive MCAD) | Systemic mast cell activation syndrome (MCAS) |
| Subvariants | - Indolent SM - Smoldering SM - Well-differentiated indolent SM - Aggressive SM - SM with associated hematologi­c neoplasm - Mast cell leukemia | - With hyper­tryptas­emia - With normal tryptas­e level |
