# Supplementary material for: Adrenal insufficiency is a contraindication for omalizumab therapy in mast cell activation disease: risk for serum sickness
Source: Naunyn Schmiedebergs Arch Pharmacol. 2020 May 6;393(9):1573–80. doi: 10.1007/s00210-020-01886-2 (PMC7419348; doi:10.1007/s00210-020-01886-2)
Supplement: Supplementary file 2 — (DOCX 26 kb) [file 210_2020_1886_MOESM2_ESM.docx]

**Table 2** Selected symptoms and signs in systemic mast cell activation disease (extract from Afrin 2013)

| **System** | **Potential manifestations of mast cell activation disease** |
| --- | --- |
| **Constitutional** | Fatigue, asthenia, weight gain or loss, generalized pruritus, environmental sensitivities (often odd) |
| **Dermatologic** | Hives, flushing, itching (with or without rash), swelling; hemangiomas (which can swell during attacks), angioedema, subcutaneous nodules; dermatographism |
| **Ophthalmologic** | Irritated eyes, increased or decreased tearing, difficulty focusing, conjunctivitis |
| **Otologic** | Tinnitus, hearing loss or hyperacusis |
| **Oral/oropharyngeal** | Painful sores, burning, throat tickle, discomfort, irritation and pain; apthous ulcers, pharyngitis, dental decay |
| **Lymphatic** | Swollen glands, lymphadenopathy, splenomegaly |
| **Pulmonary** | Difficulty taking a deep breath, cough, wheezing, nasal congestion, postnasal drip, sinus pressure; rhinitis, sinusitis; bronchitis; asthma |
| **Cardiovascular** | Lightheadedness, dizziness, vertigo, syncope, hypertension and/or hypotension, palpitations, dysrhythmias, chest discomfort or pain, hemangiomas, migratory edema (in spite of normal cardiac and renal function) |
| **Gastrointestinal** | Pain/inflammation (often migratory) in one or more segments of the luminal tract (from esophagitis to proctitis), nausea, diarrhea and/or constipation (often alternating), malabsorption, angioedema in any segment of the luminal tract, |
| **Genitourinary** | Frequency, urgency, pelvic pain, irregular and/or heavy menses; endometriosis, interstitial cystitis, chronic kidney disease |
| **Musculoskeletal** | Muscle pain and tenderness (often migratory, joint pain, bone pain; muscle tenderness by palpation, subclinical myositis (i.e., asymptomatic elevated creatine kinase), arthritis (typically migratory), osteoporosis/osteopenia, osteosclerosis, sometimes mixed |
| **Neurologic** | Headache, migraine, numbness, burning or tingling paresthesias, restless, uncomfortable legs at night |
| **Psychiatric** | Anxiety, depression, panic attacks, memory difficulties (brain fog – word finding difficulties), sleep disruptions, numerous psychiatric findings |
| **Endocrinologic/**  **Metabolic** | Abnormal electrolytes (often calcium) and liver function tests, hypothyroidism, dyslipidemia, hyperferritinemia |
| **Hematologic/**  **Coagulopathic** | Easy bruising and aberrant bleeding, polycythemia or anemia, leukocytosis or leukopenia, chronic (usually mild) monocytosis or eosinophilia or basophilia, thrombocytosis or thrombocytopenia, arterial and/or venous thromboembolic disease, |
| **Immunologic** | Multiple sensitivities to drugs and excipients, marked reactions to insect bites, impaired healing; hives, angioedema, keloids, concomittant autoimmune diseases, altered immunoglobulin levels, increased risk for cancer |

Afrin L. Presentation, diagnosis, and management of mast cell activation syndrome. In: Murray D, ed. Mast cells: phenotypic features, biological functions, and role in immunity, pp. 155–231. Happauge, NY: Nova Science Publishers, 2013.
