# Supplementary material for: Adrenal insufficiency is a contraindication for omalizumab therapy in mast cell activation disease: risk for serum sickness
Source: Naunyn Schmiedebergs Arch Pharmacol. 2020 May 6;393(9):1573–80. doi: 10.1007/s00210-020-01886-2 (PMC7419348; doi:10.1007/s00210-020-01886-2)
Supplement: Supplementary file 3 — (DOCX 25 kb) [file 210_2020_1886_MOESM3_ESM.docx]

**Table 3** Selected possible serious adverse effects of omalizumab

| **Serious adverse effect** | **Reference** |
| --- | --- |
| Anaphylaxis | Corren et al. 2009; Cox et al. 2007; Baker et al. 2016; Liebermann et al. 2017; Prescribing information |
| Serum sickness (type III allergy) | This article; Prescribing information |
| Churg-Strauss-Syndrome | Ruppert et al. 2008; Prescribing information |
| Thromboembolism | Ali 2012; Ali & Hartzema; Oblitas et al. 2019; Prescribing information |
| Atrial fibrillation | Cildag et al. 2019 |
| Cancer | Corren et al. 2009 |
| Thrombocytopenia | Prescribing information |

Ali AK (2012) Assessing the association of omalizumab use and arteriothrombotic events through spontaneous adverse event reporting. Value in Health 15: A51

Ali AK, Hartzema AG (2012) Assessing the association between omalizumab and arteriothrombotic events through spontaneous adverse event reporting. J Asthma Allergy 5:1-9

Baker DL, Nakamura GR, Lowman HB, Fischer SK (2016) [Evaluation of IgE antibodies to omalizumab (Xolair®) and their potential correlation to anaphylaxis.](https://www.ncbi.nlm.nih.gov/pubmed/26340860) AAPS J 18:115-123

Cildag S (2019) Triggering atrial fibrillation after omalizumab injection in a patient with chronic spontaneous urticarial. A case report. Med Pharm Rep 92:91-93

Corren J, Casale TB, Lanier B, Buhl R, Holgate S, Jimenez P (2009) [Safety and tolerability of omalizumab.](https://www.ncbi.nlm.nih.gov/pubmed/19302249) Clin Exp Allergy 39:788-797.

Cox L, Platts-Mills TA, Finegold I, Schwartz LB, Simons FE, Wallace DV (2007) American Academy of Allergy, Asthma & Immunology/American College of Allergy, Asthma and

Immunology Joint Task Force Report on omalizumab-associated anaphylaxis. J Allergy Clin Immunol 120:1373-1377

Lieberman PL, Jones I, Rajwanshi R, Rosén K, Umetsu DT (2017) Anaphylaxis associated with omalizumab administration: Risk factors and patient characteristics. J Allergy Clin Immunol 140:1734-1736

Oblitas CM, Galeano-Valle F, Vela-De La Cruz L, Del Toro-Cervera J, Demelo-Rodríguez P. [Omalizumab as a provoking factor for venous thromboembolism.](https://www.ncbi.nlm.nih.gov/pubmed/31320796) Drug Target Insights 13:1177392819861987

Prescribing information for Xolair, Novartis Pharma, status as of June 2019

Ruppert AM, Averous G, Stanciu D, Deroide N, Riehm S, Poindron V, Pauli G, Debry C, de Blay F (2008) Development of Churg-Strauss syndrome with controlled asthma during omalizumab treatment. J Allergy Clin Immunol 121:253-254
