# Supplementary material for: Adrenal insufficiency is a contraindication for omalizumab therapy in mast cell activation disease: risk for serum sickness
Source: Naunyn Schmiedebergs Arch Pharmacol. 2020 May 6;393(9):1573–80. doi: 10.1007/s00210-020-01886-2 (PMC7419348; doi:10.1007/s00210-020-01886-2)
Supplement: Supplementary file 4 — (DOCX 29 kb) [file 210_2020_1886_MOESM4_ESM.docx]

**Table 4** Clinical examples of type III hypersensitivity

| **Disease** | **Target** | **Main effects** |
| --- | --- | --- |
| [Systemic lupus erythematosus](https://en.wikipedia.org/wiki/Systemic_lupus_erythematosus) | Nuclear antigens | - Nephritis - Skin lesions - Arthritis |
| Rheumatoid arthritis | Citrullinated proteins; IC with IgM, IgG | - Arthritis |
| [Post-streptococcal glomerulonephritis](https://en.wikipedia.org/wiki/Post-streptococcal_glomerulonephritis) | Streptococcal cell wall antigens | - Nephritis |
| [Polyarteritis nodosa](https://en.wikipedia.org/wiki/Polyarteritis_nodosa) | Hepatitis B virus [surface antigen](https://en.wikipedia.org/wiki/HBsAg) | - Systemic [vasculitis](https://en.wikipedia.org/wiki/Vasculitis) |
| [Reactive arthritis](https://en.wikipedia.org/wiki/Reactive_arthritis) | [Several bacterial](https://en.wikipedia.org/wiki/Reactive_arthritis#Causes) antigens | - Acute arthritis |
| Serum sickness | Various antigens | - Rash/urticaria - Fever - Arthralgias - Vasculitis - Nephropathy - Lymphadenopathy |
| Arthus reaction | Various antigens | - Cutaneous vasculitis |
| [Farmer's Lung](https://en.wikipedia.org/wiki/Farmer%27s_Lung) | Inhaled antigens (often mould or hay dust) | - Alveolar inflammation |
| Henoch–Schönlein purpura (IgA vasculitis) | Unknown, likely respiratory pathogen(s) | - [Purpura](https://en.wikipedia.org/wiki/Purpura) - [Glomerulonephritis](https://en.wikipedia.org/wiki/Glomerulonephritis) |
| Modified from Mitchell, Richard Sheppard; Kumar, Vinay; Abbas, Abul K.; Fausto, Nelson. Robbins Basic Pathology. Philadelphia: Saunders. ISBN 1-4160-2973-7. 8th edition. | | |
