# Supplementary material for: Adrenal insufficiency is a contraindication for omalizumab therapy in mast cell activation disease: risk for serum sickness
Source: Naunyn Schmiedebergs Arch Pharmacol. 2020 May 6;393(9):1573–80. doi: 10.1007/s00210-020-01886-2 (PMC7419348; doi:10.1007/s00210-020-01886-2)
Supplement: Supplementary file 5 — (DOCX 28 kb) [file 210_2020_1886_MOESM5_ESM.docx]

**Table 5** Frequency of occurrence of serum sickness for selected antibodies reported in the literature.

| **Monclonal antibody** | **Approved indication** | **Antibody format** | **Antigen** | **Number of pts. treated with the antibody in whom side effects occurred** | **Number (%) of pts. in whom serum sick­ness occurred** | **Simultaneously applied drugs affecting adrenal gland function** |
| --- | --- | --- | --- | --- | --- | --- |
| Mepolizumab | severe refractory eosinophilic asthma | humanized, IgG1κ | IL-5 | n/a n/a 6455 | +^2^ 5^EMA^ 6 (0.09%)^FDA^ 0^5^ |  |
| Reslizumab | severe refractory eosinophilic asthma | humanized, IgG1κ | IL-5 |  | 0^3,4, FDA^ |  |
| Benralizumab | eosinophilic asthma | humanized, IgG1κ | IL-5Rα | 2625 n/a | 4 (0.15%)^FDA^ 6^EMA^ |  |
| Infliximab | Crohn’s disease and rheumatoid arthritis | chimeric, IgG1 | TNFα | 150682 202 | 433 (0.3%)^FDA^ 11^10^ 1^6,7^ 2,8%^1^ | prednisone 5 mg |
| Alemtuzumab | B-cell chronic lymphocytic leukemia | humanized, IgG1 | CD52 | 6420 | 2 (0.03%)^FDA^ 1^EMA^ 1^8^ |  |
| Adalimumab | Crohn’s disease and rheumatoid arthritis | human, IgG1 | TNFα | n/a 520128 62 61 | +^1^  113 (0.02%)^FDA^ 0^9^ 1 (7%)^10^ |  |
| Rituximab | CD20-positive B-cell non-Hodgkin’s lymphoma | chimeric, IgG1 | CD20 | 43932 | 158 (0,36%)^FDA^ 1 ^multiple case reports11- 20^ |  |
| Cetuximab | Metastatic colorectal and head and neck  carcinoma | chimeric, IgG1 | EGRF | n/a 16351 | 1^EMA^ 3 (0,02%)^FDA^ |  |
| Panitumumab | Metastatic colorectal carcinoma | human, IgG2 | EGFR | n/a | 1^EMA^ 0^FDA^ |  |
| Tocilizumab | rheumatoid arthritis ; systemic juvenile idiopathic arthritis | human, IgG1 | IL-6R | n/a 45123 | 2^EMA^ 5 (0,01%)^FDA^ |  |

^1^ Scherer K, Spoerl D, Bircher AJ (2010) Adverse drug reactions to biologics. J Dtsch Dermatol Ges 8:411-426

^2^ Jackson K, Bahna SL (2020) Hypersensitivity and adverse reactions to biologics for asthma and allergic diseases. Expert Rev Clin Immunol 16:311-319

^3^ Molina Health care, Cinqair (reslizumab) Policy Number: C9761-A

^4^ APPLICATION NUMBER: 761033Orig1s000

^5^ Ortega HG, Meyer E, Brusselle G, Asano K, Prazma CM, Albers FC, Mallett SA, Yancey SW, Gleich GJ. Update on immunogenicity in severe asthma: Experience with mepolizumab. J Allergy Clin Immunol Pract 7:2469-2475

^6^ Ariane M, Bouaziz JD, de Masson A, Jachiet M, Bagot M, Lepelletier C (2019) Efficacy and safety of etanercept for postoperative pyoderma gangrenosum after infliximab serum sickness. Dermatol Ther 32:e12774

^7^ Marc Scherlinger, Thierry Schaeverbeke, Marie-Elise Truchetet, for the Fédération Hospitalo Universitaire ACRONIM (2017) Serum sickness-like disease after switching to biosimilar infliximab. Rheumatology 56:2032–2034

^8^ Lapucci C, Gualandi F, Mikulska M, Palmeri S, Mancardi G, Uccelli A, Laroni A (2018) Serum sickness (like reaction) in a patient treated with alemtuzumab for multiple sclerosis: A case report. Mult Scler Relat Disord 26:52-54

^9^ Moots RJ, Xavier RM, Mok CC, Rahman MU, Tsai WC, Al-Maini MH, Pavelka K, Mahgoub E, Kotak S, Korth-Bradley J, Pedersen R, Mele L, Shen Q, Vlahos B (2017) The impact of anti-drug antibodies on drug concentrations and clinical outcomes in rheumatoid arthritis patients treated with adalimumab, etanercept, or infliximab: Results from a multinational, real-world clinical practice, non-interventional study. PLoS One 12:e0175207

^10^ Lees CW, Ali AI, Thompson AI, Ho GT, Forsythe RO, Marquez L, Cochrane CJ, Aitken S, Fennell J, Rogers P, Shand AG, Penman ID, Palmer KR, Wilson DC, Arnott ID, Satsangi J (2009) The safety profile of anti-tumour necrosis factor therapy in inflammatory bowel disease in clinical practice: analysis of 620 patient-years follow-up. Aliment Pharmacol Ther 29:286-297

^11^ Holmøy T, Fogdell-Hahn A, Svenningsson A (2019) Serum sickness following rituximab therapy in multiple sclerosis. Neurol Clin Pract 9:519-521

^12^ Podestà MA, Ruggiero B, Remuzzi G, Ruggenenti P (2020) Ofatumumab for multirelapsing membranous nephropathy complicated by rituximab-induced serum-sickness. BMJ Case Rep 13(1).

^13^ Nakamura M, Kanda S, Yoshioka Y, Takahashi C, Owada K, Kajiho Y, Harita Y, Oka A (2020) Rituximab-induced serum sickness in a 6-year-old boy with steroid-dependent nephrotic syndrome. CEN Case Rep 9:173-176

^14^ Wolf AB, Ryerson LZ, Pandey K, McGettigan BM, Vollmer T, Corboy JR, Alvarez E (2019) Rituximab-induced serum sickness in multiple sclerosis patients. Mult Scler Relat Disord 36:101402.

^15^ Bayer G, Agier MS, Lioger B, Lepelley M, Zenut M, Lanoue MC, Maillot F, Jonville-Bera AP (2019) Rituximab-induced serum sickness is more frequent in autoimmune diseases as compared to hematological malignancies: A French nationwide study. Eur J Intern Med 67:59-64

^16^ Mantilla B, Liew JW (2018) Avoiding a rash diagnosis: rituximab-induced serum sickness. J Clin Rheumatol, doi: 10.1097/RHU.0000000000000918

^17^ Vendramin C, Thomas M, Westwood JP, McGuckin S, Scully M (2019) Rituximab-induced acute and delayed serum sickness in thrombotic thrombocytopenic purpura: the role of anti-rituximab antibodies. Br J Haematol 184:858-861

^18^ Cheng DR, Silverman ED, Cho R. (2020) Coughing up clues: 16-year-old girl with acute haemoptysis. BMJ Case Rep 13(1)

^19^ Todd D, Helfgott S (2007) Serum sickness following treatment with rituximab. J Rheumatol 34:430-433

^20^ Sandhu A, Harford A, Singh P, Alas E (2012) Is thymoglobulin or rituximab the cause of this serum sickness? A case report of serum sickness dilemma and literature review. Case Rep Med 2012:234515.
